# Effect of adding a psychological intervention to routine care of common mental disorders in a specialized mental healthcare facility in Pakistan: a randomized controlled trial

**DOI:** 10.1186/s13033-020-00434-y

**Published:** 2021-01-19

**Authors:** Syed Usman Hamdani, Zill-e- Huma, Aqsa Masood, Kaina Zhou, Zainab Ahmed, Huma Nazir, Hania Amin, Parveen Akhtar, Richard A. Bryant, Katie Dawson, Mark van Ommeren, Duolao Wang, Atif Rahman, Fareed Aslam Minhas

**Affiliations:** 1 University of Liverpool, Liverpool, UK; 2 Human Development Research Foundation, Islamabad, Pakistan; 3 Liverpool School of Tropical Medicine, Liverpool, UK; 4 Institute of Psychiatry, WHO Collaborating Center for Mental Health Research and Training, Rawalpindi, Pakistan; 5 University of New South Wales, Sydney, Australia; 6 World Health Organization, Geneva, Switzerland

**Keywords:** Low intensity psychological intervention, Cognitive behavioral therapy, Specialized mental health facility, Common mental disorders, Depression, Anxiety

## Abstract

**Background:**

In many low resource settings, the provision of government mental health care services is limited to specialized psychiatry units in urban hospital care facilities, where the most common treatment for common mental disorders (CMDs) is pharmacotherapy, occasionally with adjunct nonspecific psychological support. We aimed to evaluate the effectiveness of adding a low intensity, psychological intervention, Problem Management Plus (PM+) for CMDs into routine care in a specialized mental health care facility in Pakistan.

**Methods:**

A two arm, single-blind individual randomized controlled trial (RCT) was carried out with adults (N = 192), referred for psychological support by psychiatrists. The study participants were randomized (1:1) to PM + plus Treatment as Usual (TAU) (n = 96) or TAU only (n = 96). The primary outcomes were symptoms of anxiety and depression, measured by the Hospital Anxiety and Depression Scale (HADS) and functional impairment as measured by WHO Disability Assessment Schedule (WHODAS 2.0) at 20 weeks after baseline.

**Results:**

The analysis was done on intention-to-treat principle. The linear mixed model analysis showed that at 20 weeks after baseline, there was a significant reduction in symptoms of anxiety and depression (mean [SD], 16.23 [8.81] vs 19.79 [7.77]; AMD, − 3.10; 95% CI, − 0.26 to − 5.76); *p* = 0.03 and improvement in functioning (mean [SD], 22.94 [9.37] vs 27.37 [8.36]; AMD, − 4.35; 95% CI, − 1.45 to − 7.24); *p* = 0.004 in PM + plus TAU versus TAU arm. The follow-up rate was 67% at primary end-point.

**Conclusions:**

Specialized care facilities in LMICs may consider adding brief, evidence-based psychological treatments for CMDs to their routine care.

*Trial Registration* Australian New Zealand Clinical Trials Registry, ACTRN12616000381482. Registered March 23, 2016. Retrospectively registered, https://www.anzctr.org.au/Default.aspx/ ACTRN12616000381482

## Background

Common mental disorders (CMDs), such as anxiety and depression, are one of the leading causes of disability globally [1]. In low and middle-income countries (LMICs), the treatment gap for CMDs is more than 90% [2]. Despite the wide-spread recognition of this gap for CMDs in LMICs, the provision of government mental health care services is usually limited to specialized psychiatry units, located in hospital care facilities [3]. Mental health needs are evident in Pakistan, which has been affected by chronic adversity in the form of socio-political instability, economic uncertainty, regional conflict and dislocation. These events have led to increased levels of psychological and social suffering, including a high prevalence of mental disorders in Pakistan [4, 5].

A number of psychological intervention manuals—mainly based on cognitive behavioral and interpersonal psychotherapy—have been developed and proven effective for treating CMDs in primary health care and community settings in LMICs [6–10] and meta-analyses suggest that such interventions are as effective as pharmacotherapy [11]. The World Health Organization (WHO) recommends that both anti-depressants and psychological interventions should be available to people with depression [12]. A brief, multicomponent behavioral WHO intervention called Problem Management Plus (PM +) was developed for the management of CMDs in settings affected by adversity [13]. There have been multiple trials of PM + delivered in individual and group formats [9, 10, 14]. However, these studies have focused on evaluating the effectiveness of PM + to reduce CMDs in primary health care and community settings.

The mainstay of treatment for CMDs in many psychiatric units is pharmacotherapy [15], occasionally with adjunct nonspecific psychological support. It is thus valuable to evaluate whether evidence-based psychological interventions can be effectively added in routine specialist health context to reduce the burden of CMDs in LMICs.

## Methods

This study aimed to evaluate the effectiveness of adding PM + for CMDs to routine care in a specialized mental health care facility in Pakistan. We hypothesized that PM + in combination with Treatment-as-Usual (TAU) will be superior compared to TAU alone, to reduce the symptoms of depression and anxiety measured with Hospital Anxiety and Depression Scale (HADS) total score at 20 weeks after baseline.

### Design

We conducted a single-blind individual randomized controlled trial (RCT) to evaluate the effectiveness of PM + plus Treatment-as-Usual (TAU) versus TAU alone in the treatment of CMDs in patients presenting in the outpatient department of Institute of Psychiatry, Rawalpindi, Pakistan. The study was conducted over the duration of 12 months. Ethical approval of the study was obtained from the Institutional Review and Ethics Board of the Rawalpindi Medical University and Allied Hospitals Rawalpindi (ethical approval certificate number ERC/RMU/23/05/2015). The full trial protocol is available online [16]. Trial registration: Australian New Zealand Clinical Trials Registry, ACTRN12616000381482. Registered Retrospectively on March 23, 2016.

### Study settings

The participants were recruited from the Institute of Psychiatry (IoP), WHO Collaborating Centre for Mental Health Research and Training, Benazir Bhutto Hospital-the public sector tertiary care hospital in Rawalpindi, Pakistan. IoP is the hub of mental health policy, services, training and research in Pakistan. It is the specialist referral facility for the patients with mental health problems presenting in the primary and secondary health care facilities in the north of Pakistan. Patient services include in-patients and outpatient services which are led by teams comprising of consultant psychiatrists and psychologists, trainee psychiatrists and psychologists, social workers and interns.

### Participants

The target population for this study was adult (age 18–60 years) outpatient department attendees, referred for psychological support for depression, anxiety and stress related conditions by psychiatrists after clinical evaluation who (a) scored above 2 on a screening questionnaire for psychological distress (General Health Questionnaire-12; GHQ-12); [17, 18] and (b) scored above 16 on a screening questionnaire for functional impairments (WHO Disability Assessment Schedule – WHODAS 2.0) [19]. As PM + is not suitable for the treatment of severe mental health problems (including psychosis or risk of suicide) [13], participants with imminent risk of suicide, severe mental disorder (e.g. psychotic disorders, substance dependence), or severe cognitive impairment (e.g. severe intellectual disability, dementia) as assessed by psychiatrists, were excluded from the study.

### Interventions

#### Problem Management plus (PM+)

The WHO PM + intervention (individual version) [13] is based on established principles of problem solving and behavioral techniques and is delivered in five weekly face-to-face sessions administered on an individual basis. The average duration of each session is 90 min. The two key features of WHO PM + program are (a) the program takes a task-shifting approach where non-specialists (workers without a professional license specific for mental health care such as, in Pakistan, psychology graduates, nurses and community volunteers) can deliver this program to adults experiencing common mental health problems (e.g. anxiety, stress, depression and grief) and (b) it is trans-diagnostic—it addresses a range of symptoms for common mental disorders and does not require an expert diagnosis of condition to qualify treatment. PM + providers in this study had a master’s degree (16 years of education) in psychology and received eight days training in PM + by the master trainer followed by fortnightly supervision meetings with the master trainer.

#### Treatment‑as‑Usual (TAU)

Treatment-as-usual in the outpatient department consists of an initial assessment by trainee psychiatrists followed by an expert consultation on the case by consultant psychiatrist. The main stay of treatment is pharmacotherapy and psychological support where needed. A psychological support session comprises of brief semi-structured psycho-education sessions for patients with depression and anxiety symptoms and training in anger and stress management strategies such as breathing exercises. A complete record of services received by trial participants in both study arms was obtained using the Client Services Receipt Inventory (CSRI).

#### Primary outcomes

The primary outcomes were (a) symptoms of anxiety and depression measured using the Hospital Anxiety and Depression Scale (HADS) [20, 21] and (b) functional impairment as measured by WHODAS 2.0 [22] at 20 weeks after baseline.

- The HADS is a well-established 14-item scale consisting of two subscales: HADS-A (anxiety, seven items, and scores range from 0–21) and HADS-D (depression, seven items, scores range from 0–21), where higher scores indicate more anxiety and/or depression. In our study, we used a previously validated Urdu version of HADS [20].
- Functional impairment was assessed using the 12-item WHO Disability Assessment Schedule (WHODAS 2.0) [22]. WHODAS 2.0 assesses participants’ health-related difficulties in the level of functioning in six domains of life (understanding and communicating; moving and getting around; attending to one’s hygiene, dressing, eating and staying alone; interacting with other people; domestic responsibilities, leisure, work and school and joining in community activities, participating in society) over the past 30 days. WHODAS 2.0 has been extensively used in different populations and health conditions including mental health and established itself as gold standard to measure functioning [23].

All the study tools were administered in local (i.e. Urdu) language and have been previously used in the similar settings of Pakistan [9, 10, 24].

An independent assessment team, trained in the ethical conduct of research and assessments and blinded to the allocation status of the trial participants, conducted baseline and post-intervention assessments.

#### Secondary outcomes

The secondary clinical outcomes included;

The Patient Health Questionnaire-9 (PHQ-9), which is a 9-item instrument measuring presence and severity of depression during the past 2-weeks on a 4-point Likert scale ranging from ‘*not at all*’ to ‘*nearly every day*’. The PHQ-9 total severity score ranges from 0 to 27 [25]. Higher scores indicate more severe depression. We used the previously validated Urdu version of PHQ-9 [26, 27].

The 17 item Post Traumatic Stress Symptoms-Checklist (PCL-C) was used to measure DSM-IV based posttraumatic stress disorder symptoms. The participants are asked to rate their responses on a 5-point scale during the past week. The PCL-C has been used previously in Pakistan [28] and found to have good psychometric properties (Mushtaq, unpublished data, 2013). In the current study, we changed the reporting time of PCL from last month to last week to enhance the sensitivity of tool to detect change at the 3 months’ post-intervention.

Psychological profile outcome (PSYCHLOPS)—[29] assesses personally identified problems. It consists of four questions that encompass three domains: problems (2 questions), functioning (1 question) and wellbeing (1 question). Participants are asked to give free text responses to the problem and function domains. Responses are scored on an ordinal 6-point scale producing a maximum score of 20 (6 points per domain). The PSYCHLOPs version administered at post-treatment and follow-up also includes an overall evaluation question (determining self-rated outcome ranging from ‘*much better*’ to ‘*much worse*’) in relation to the identified problem. PSYCHLOPS has been validated in primary care populations across several countries [30, 31].

The Multidimensional Scale of Perceived Social Support (MSPSS) [32] aims to measure perceived social support. It includes 12 items which cover three dimensions: support from family, friends and significant other. Each item is rated on a 7-point Likert-scale (1 = *very strongly disagree*; 7 = *very strongly agree*). A total score is calculated by summing the responses of all items (range 12–84) with higher scores indicating higher levels of perceived social support. The MSPSS has been validated in Urdu [33].

Services accessed by participants were recorded using the Client Services Receipt Inventory (CSRI). The CSRI was developed for the collection of data on service utilization and related characteristics of people with mental disorders, as the basis for calculating the costs of care for mental health cost-effectiveness research [34]. It has been previously used in Pakistan and India [34, 35].

#### Procedure

Patients who presented at the outpatient department between July 2015 and November 2016 for treatment of CMDs were evaluated by a psychiatrist. After clinical evaluation, if the psychiatrist decided psychological support was indicated, s/he introduced the study and took verbal consent for participation in the study. Written informed consent was obtained from eligible participants by the study coordinator. Participants were randomized to intervention or control arm on a 1:1 basis using computerized software by an independent researcher. The allocation concealment was ensured by keeping random sequence in sequentially numbered, opaque, sealed envelopes, at the off-site center. The intervention arm participants received a total of five weekly individual sessions of PM + program by PM + providers and TAU from the psychiatrists/psychologists. The control arm participant received TAU from the psychiatrist/psychologists at the mental health facility. Post-treatment assessments were carried out at two-time points a) seven-weeks after baseline and b) 20 weeks after baseline. Participants who developed severe psychiatric problems (e.g., psychosis, imminent suicidality) at follow-up were referred to psychiatrists for specialist care (n = 3).

### Statistical analysis

Sample size calculation was based on a multicenter study of culturally-adapted cognitive behavioral therapy (CBT)-based intervention conducted in Pakistan that used the HADS as the primary outcome measure [36]. A two-point reduction in HADS depression score between the intervention and control arm is considered to be clinically relevant [36]. With *p* < 0.05 and 90% power, a total of 96 participants were needed. Accounting for an expected drop-out rate of 50%, the total sample size was 192 participants, who were equally randomized to PM + (n = 96) and TAU (n = 96).

The data was analyzed using an Intent-To-Treat (ITT) analysis approach. The primary outcome was summarized using number of participants (n), means and standard deviations (SD). To estimate the treatment effect, a linear mixed model was employed for the primary endpoint analysis, which had treatment, visit, interaction between treatment and visit as fixed effects, baseline measurement of primary endpoint as covariate, and subject as random effects. The mean difference between two treatment arms at each visit/time together with its 95% confidence interval was derived from the mixed model. Covariate-adjusted mixed model of primary endpoint (20 weeks after baseline) was also performed by adding pre-specified covariates at baseline into the above model. Secondary continuous outcomes were analyzed in a similar way. Missing data was treated as missing at random in the mixed model analysis and no imputation of primary and secondary endpoints was made. All analyses were performed using SAS 9.3.

## Results

**Fig. 1:**
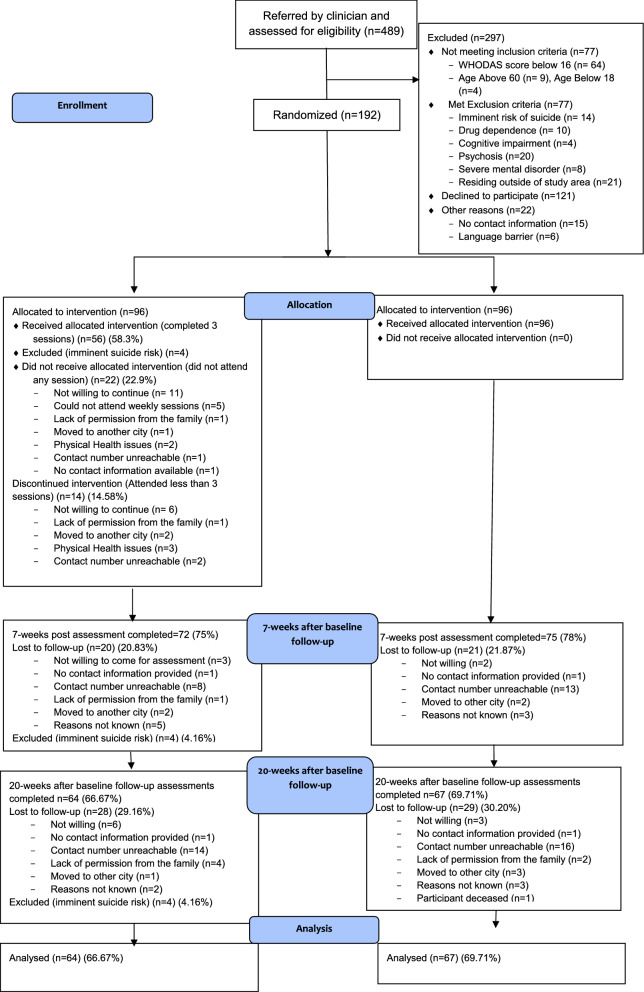
Participants’ flow

489 patients were assessed for eligibility. 192 participants, who met the inclusion criteria and provided informed consent were enrolled in the trial and randomly allocated (1:1) to PM + plus TAU (n = 96) and TAU arms (n = 96) (Fig. 1). The follow-up rate for primary outcome at the primary endpoint (20 weeks after baseline) was 64.5% (62 of 96 participants) in the intervention arm and 69.79% (67 of 96 participants) in the control arm. There were no significant differences between the two arms in demographic characteristics and symptom scores at baseline. The mean (± SD) age of the participants was 34.05 (± 10.47) years. 68% (128/192) of the participants were females (Table 1).

**Table 1:** Demographic characteristics of the study participants (*n*, %)

Variable	PM + N (%) (n = 96)	TAU N (%) (n = 96)	Total (N = 192)
Age (mean ± SD)	33.03 ± 9.94	35.07 ± 10.95	34.05 ± 10.47
Gender			
Male	32 (33.3)	28 (29.2)	60(31.9)
Female	62 (64.6)	66 (68.8)	128 (68)
Education			
Uneducated	15 (15.6)	25 (26)	40 (20.8)
Completed primary (grade 5)	20 (20.8)	12 (12.5)	32 (16.7)
Completed middle (grade 8)	9 (9.4)	13 (13.5)	22(11.5)
Completed matriculate (grade 10)	16 (16.7)	14 (14.6)	30 (15.6)
Completed College and University (grade 11–16)	36 (37.5)	30 (31.3)	66 (34.4)
Marital status			
Never married	28 (29.8)	19 (19.8)	47(24.5)
Currently married	61 (63.5)	69 (71.9)	130 (67.7)
Separated	1 (1.0)	2 (2.1)	3 (1.6)
Divorced	3 (3.1)	2 (2.1)	5 (2.6)
Widowed	3 (3.1)	4 (4.2)	7 (3.6)
Employment status			
Employed	31 (32.3)	26 (27.1)	57 (29.7)
Not employed	65 (67.7)	70 (72.9)	135 (70.3)
Occupation			
Paid work	24 (25.8)	20 (21.3)	44 (22.9)
Self-employed	10 (10.8)	8 (8.5)	18(9.4)
Student	2 (2.2)	3 (3.2)	5(2.6)
Keeping house/homemaker	14 (15.1)	18 (19.1)	32 (16.7)
Retired	0 (0.0)	2 (2.1)	2 (1)
Unemployed (health reasons)	2 (2.2)	1 (1.1)	3 (1.6)
Unemployed (other reasons)	41 (44.1)	42 (44.7)	83 (43.2)
Living structure			
Nuclear	49 (51.0)	47 (49.5)	96(50)
Joint/extended	47 (49.0)	48 (50.5)	95 (49.7)

58% of participants attended 3 or more sessions of PM + intervention. The mean number of intervention sessions attended by trial participants was 2.84 (± 2.16). Each session lasted for an average of 90 min. The quality of intervention delivery was assessed by completing a self-check for each session by the PM + providers, supported by peer-supervision and fortnightly supervisions with the mental health specialist via Skype. In terms of medication, 36/64 (56%) participants in PM + and 49/67 (73%) participants in TAU reported receiving a prescription of psychotropic medicines.(Table 2).

**Table 2:** Health services utilization across two arms after 20-weeks after baseline

Health service providers accessed	PM+ ( *f)*	TAU ( *f)*
Psychiatrist	45	49
Specialist doctor	21	33
General doctor	16	11
Pharmacist	1	2
Traditional healer	2	1
Other mental health worker	2	–
Number of visits		
3 visits	8	13
2 visits	30	32
1 visit	54	65
Location		
Public hospital	47	53
Private hospital	11	6
Local health centre	–	5
Reasons for visit		
Follow-up visit for treatment of depression or anxiety problems	50	60
Sleep problems	10	13
Acute aggravation of mental health problems	6	13
Other mental health problems e.g. maternal/perinatal mental health condition	15	16
Infectious diseases and injuries	2	2
Medications		
A. Antidepressants (n = 58)		
SSRIs	22	31
TCAs	2	3
B. Anxiolytics	8	9
C. Mood stabilizers	2	1
D. Anti-psychotics	2	5
E. Multi-vitamins	8	5
F. Analgesics	4	3
G. Others (anti- hypertensive, anti-diabetic, thyroid related)	6	9
Average duration of consultation (in minutes) M(SD); min–max	7.62 (± 2.57); 3–15	8.85 (± 3.99); 2–20
Average consultation fee paid (in PKR) M(SD); min–max	32 (± 129.9): 0–700	3.10 (± 14.79); 0–100

Due to multiplicity of contacts with service providers and treatments received, the table reports frequency of contacts and prescribed treatment; 3 in intervention and 2 participants in TAU were hospitalized during the study

Seven-weeks after baseline, the intervention arm had significantly lower scores on HADS (mean [SD], 16.72[8.44] vs 21.0 [7.88]; adjusted mean difference [AMD], 4.35; 95% CI, 1.71 to 6.99). At 20 weeks after baseline, the intervention arm had significantly lower scores than the control arm on HADS (mean [SD], 16.23 [8.81] vs 19.79 [7.77]; AMD, 3.10; 95% CI, 0.26 to 5.76). Seven-weeks after baseline, the intervention arm had significantly lower scores on HADS-A (mean [SD], 8.56 [5.00] vs 11.08 [4.90]; AMD, 2.41; 95% CI, 0.77 to 4.04) and HADS-D (mean [SD], 8.16 [4.17] vs 9.92 [4.37]; AMD, 1.85; 95% CI, 0.41 to 3.29). At 20 weeks after baseline, the intervention arm had significantly lower scores than the control arm on HADS-A (mean [SD], 8.17 [5.12] vs 10.12 [4.79]; AMD, 1.69; 95% CI, − 0.02 to 3.41), however, there was no significant difference between two arms on HADS-D (mean [SD], 8.04 [4.89] vs 9.67 [3.94]; AMD, 1.30; 95% CI, − 0.21 to 2.81).(Table 3).

**Table 3:** Summary statistics and results from mixed-model analysis of primary and secondary outcomes

Primary and secondary outcomes	Visit	PM + (N = 96)		TAU (N = 96)		Difference in least squares mean (95% CI)	*P*-value
		N	M (SD)	N	M (SD)		
Descriptive statistics n, mean (SD)							
HADS Anxiety	Pre-treatment	95	13.44 (3.67)	96	12.90 (4.02)		
	Post-treatment	73	8.56 (5.00)	73	11.08 (4.90)	− 2.41 (− 4.04 to − 0.77)	0.004
	Follow-up	62	8.17 (5.12)	67	10.12 (4.79)	− 1.69 (− 3.41 to 0.02)	0.05
HADS Depression	Pre-treatment	95	12.18 (3.99)	96	10.80 (3.72)		
	Post-treatment	73	8.16 (4.17)	73	9.92 (4.37)	− 1.85 (− 3.29 to − 0.41)	0.01
	Follow-up	62	8.04 (4.89)	67	9.67 (3.94)	− 1.30 (− 2.81 to 0.21)	0.09
HADS	Pre-treatment	95	25.62 (6.61)	96	23.70 (6.07)		
	Post-treatment	73	16.72 (8.44)	73	21.00 (7.88)	− 4.35 (− 6.99 to − 1.71)	0.002
	Follow-up	62	16.23 (8.81)	67	19.79 (7.77)	− 3.10 (− 5.76 to − 0.26)	0.03
WHODAS	Pre-treatment	96	33.41 (7.17)	96	32.77 (7.77)		
	Post-treatment	74	25.20 (8.97)	74	28.41 (8.19)	− 3.16 (− 6.38 to − 0.85)	0.01
	Follow-up	62	22.94 (9.73)	67	27.37 (8.36)	− 4.35 (− 7.24 to − 1.45)	0.004
PCL	Pre-treatment	96	49.78 (13.99)	96	49.86 (13.66)		
	Post-treatment	61	39.90 (15.89)	63	42.35 (15.73)	− 2.69 (− 8.08 to 2.70)	0.32
	Follow-up	61	32.93 (14.21)	64	39.08 (15.75)	− 5.39 (− 10.76 to − 0.02)	0.05
PHQ	Pre-treatment	96	15.09 (5.17)	96	15.04 (4.99)		
	Post-treatment	53	9.79 (6.71)	54	11.76 (6.51)	− 2.18 (− 4.65 to 0.29)	0.08
	Follow-up	61	7.16 (6.23)	63	10.29 (6.79)	− 2.91 (− 5.22 to − 0.60)	0.01
Days of these difficulties present	Pre-treatment	92	20.9 3 (9.61)	93	20.89 (8.51)		
	Post-treatment	66	13.98 (10.45)	67	18.10 (10.21)	− 3.93 (− 7.44 to − 0.43)	0.03
	Follow-up	60	13.88 (11.17)	64	16.67 (9.59)	− 2.80 (− 6.40 to 0.80)	0.13
Days of total disability	Pre-treatment	91	11.22 (9.78)	92	10.82 (9.47)		
	Post-treatment	65	5.03 (7.15)	67	7.82 (9.03)	− 2.62 (− 5.29 to 0.06)	0.06
	Follow-up	60	5.82 (7.34)	64	8.00 (7.82)	− 1.71 (− 4.46 to 1.03)	0.22
Days of activity reduce	Pre-treatment	90	14.14 (10.26)	92	12.97 (10.19)		
	Post-treatment	65	8.22 (8.40)	67	11.19 (9.58)	− 2.72 (− 5.92 to 0.49)	0.10
	Follow-up	60	8.35 (9.57)	64	10.20 (9.69)	− 1.92 (− 5.22 to 1.38))	0.25
Perceived social support	Pre-treatment	96	51.92 (16.60)	96	52.74 (17.11)		
	Post-treatment	73	55.53 (17.70)	74	51.43 (18.03)	3.85 (− 4.55 to 9.25)	0.16
	Follow-up	61	56.61 (17.35)	65	52.57 (14.70)	2.24 (− 3.42 to 7.90)	0.43
PSYCHLOPS	Pre-treatment	96	15.39 (3.65)	95	14.94 (3.77)		
	Post-treatment	67	9.31 (5.68)	69	11.55 (5.01)	− 2.40 (− 4.19 to − 0.60)	0.009
	Follow-up	61	8.98 (5.93)	61	10.62 (4.99)	− 1.63 (− 3.51 to 0.24)	0.09

*TAU* Treatment as usual, *PM* + Problem Management Plus, *HADS* Hospital Anxiety and Depression Scales (subscale score range: 0–21; higher scores indicate elevated anxiety or depression, respectively), *WHODAS* WHO Disability Adjustment Scale (total score range: 0–48; higher scores indicate more severe impairment), *PCL* Posttraumatic Stress Disorder Checklist (total score range: 17–85; higher scores indicate more severe PTSD severity), *PHQ* Patient Health Questionnaire (total score range: 0–27; higher scores indicate more severe depression, *PSYCHLOPS* Psychological Outcome Profiles (total score range: 0–20; higher scores indicate poorer outcome)

Post-treatment = 7-week after baseline; Follow-up = 20 weeks after baseline

Seven-weeks after baseline, the intervention arm had significantly lower scores on WHODAS 2.0 (mean [SD], 25.20[8.97] vs 28.41 [8.19]; AMD, 3.16 95% CI, 0.85to 6.38). At 20 weeks after baseline, the difference of WHODAS 2.0 scores between intervention arm and control arm was sustained (mean [SD], 22.94 [9.37] vs 27.37 [8.36]; AMD, 4.35; 95% CI, 1.45 to 7.24).

At 20 weeks after baseline, there was a significant reduction in the level of posttraumatic stress in the intervention arm (mean [SD], 32.93[14.21] compared to the control arm 39.08 [15.75]; AMD, 5.39; 95% CI, 0.02 to 10.76). Similarly, there was significant reduction in the symptoms of depressive disorder in the intervention arm (PHQ-9: mean [SD], 7.16[6.23] compared to the control arm 10.29[6.79]; AMD, 2.91; 95% CI, 0.60 to 5.22) at 20 weeks after baseline. However, no significant differences were observed in the scores of PSYCHLOPS (mean[SD], 8.98(5.93) vs 10.62[4.99]; AMD, 1.63; 95% CI, − 0.24 to 3.51), and social support (mean[SD], 56.61[17.35] vs 52.57[14.70]; AMD, − 2.24; 95% CI, − 7.90 to 3.42) between the two arm at 20 weeks after baseline.

The findings from covariate adjusted analysis showed that, after controlling for the baseline measurements (i.e. age, gender, severity on HADS), there was significant improvement in the intervention arm than TAU arm at both seven and 20 weeks after baseline in the clinical outcome measures.

(Table 4).

**Table 4:** Summary results from post hoc mixed-model analysis of primary and secondary outcomes, with baseline measurement (i.e. age, gender, HADS) included as covariate

Primary and secondary outcomes	Visit	Difference in least squares mean for intervention vs treatment as usual (95% CI)	*P *value
HADS Anxiety	Pre-treatment		
	Post-treatment	− 2.67 (− 4.32 to − 1.03)	0.002
	Follow-up	− 1.84 (− 3.56 to − 0.13)	0.04
HADS Depression	Pre-treatment		
	Post-treatment	− 1.98 (− 3.41 to − 0.55)	0.007
	Follow-up	− 1.43 (− 2.92 to 0.07)	0.06
HADS	Pre-treatment		
	Post-treatment	− 4.65 (− 7.27 to − 2.03)	0.0006
	Follow-up	− 3.20 (− 5.92 to − 0.48)	0.02
WHO DAS	Pre-treatment		
	Post-treatment	− 3.81 (− 6.59 to − 1.04)	0.008
	Follow-up	− 4.44 (− 7.34 to − 1.55)	0.003
PCL	Pre-treatment		
	Post-treatment	− 3.30 (− 8.71 to 2.12)	0.23
	Follow-up	− 5.17 (− 11.08 to − 0.33)	0.04
PHQ	Pre-treatment		
	Post-treatment	− 2.51 (− 4.96 to − 0.06)	0.04
	Follow-up	− 3.04 (− 5.32 to − 0.76)	0.01
Days of these difficulties present	Pre-treatment		
	Post-treatment	− 4.33 (− 7.90 to − 0.77)	0.02
	Follow-up	− 2.84 (− 6.47 to 0.79)	0.12
Days of total disability	Pre-treatment		
	Post-treatment	− 2.51 (− 5.18 to 0.15)	0.06
	Follow-up	− 1.69 (− 4.41 to 1.02)	0.22
Days of activity reduce	Pre-treatment		
	Post-treatment	− 3.15 (− 6.41 to 0.10)	0.06
	Follow-up	− 1.95 (− 5.27 to 1.37)	0.25
Perceived social support	Pre-treatment		
	Post-treatment	4.45 (− 1.05 to 9.95)	0.11
	Follow-up	2.52 (− 3.22 to 8.27)	0.39
PSYCHLOPS	Pre-treatment		
	Post-treatment	− 2.58 (− 4.39 to − 0.77)	0.006
	Follow-up	− 1.66 (− 3.54 to 0.23)	0.08

*HADS* Hospital Anxiety and Depression Scale, *WHODAS* WHO Disability Assessment Schedule 2.0, *PCL* Post-traumatic stress disorder checklist, *PHQ* Patient Health Questionnaire, *PSYCHLOPS* Psychological Outcome Profile

Post-treatment = 7-week after baseline; Follow-up = 20 weeks after baseline

## Discussion

The addition of PM + intervention to routine care in a specialist mental health facility in Pakistan led to improvements in symptoms of anxiety, depression, post-traumatic stress, and functioning at seven and 20 weeks after baseline compared to routine care alone. PM + involves evidence based psychological strategies (including problem solving, stress management, behavioral activation, and strengthening social support) that seek to ameliorate the symptoms of anxiety and depression [13]. It has been evaluated with adults affected by adversity and experiencing psychological distress in a number of studies conducted in low resource contexts and settings, globally [9, 10, 14, 24]. The results of the current study demonstrate that low intensity psychological intervention delivered by non-specialists, can improve the symptoms of anxiety and depression among adults in a specialist mental health care facility.

A key strength of the current study is that, we compared two active interventions for treatment of CMDs in a specialist mental health care facility in Pakistan (PM + plus routine care versus routine care alone). The results demonstrated noticeable gains in the treatment of anxiety and depression symptoms with addition of PM + to ongoing routine care that consisted of pharmacotherapy in most cases. The findings of our study are consistent with a systematic review and meta-analysis of literature by Cuijpers et al. [11], which concluded that pharmacotherapy and psychotherapy both contribute equally and independently to the effects of combined treatment. Hence, PM + may be a feasible and effective treatment option in outpatient psychiatry departments in LMICs, especially, where there are currently no defined psychological interventions available. However, further studies with placebo and combined pharmacotherapy and psychotherapy may be designed to understand the clinical advantage of combined treatment with PM + intervention in tertiary care facilities.

Another strength of this study is the finding that non-specialists can effectively deliver the PM + intervention to improve treatment outcomes for CMDs in a specialist mental health care facility in Pakistan. There is an urgent need to expand the mental health care workforce to meet the ever-increasing demand for evidence-based, holistic mental health services in low resource settings. Our study demonstrates that trans-diagnostic psychological intervention for CMDs can be delivered by non-specialists such as psychology graduates, in outpatient psychiatry departments of low resource settings. Other studies in global mental health literature have reported similar findings where nurses, community health workers and peer volunteers have effectively delivered low intensity psychological interventions in low resource settings [6, 8, 9, 36–39].

It may be expected that the study participants at the tertiary care facility in the current study would have more severe depression and anxiety symptoms than the participants with the CMDs from community samples. Yet, the participants in both study arms had baseline scores on symptoms of anxiety and depression similar to the other studies [9, 10, 14] that have evaluated the effectiveness of PM + intervention in primary care and community settings; the drop in the symptoms scores on HADS in the present study as a result of intervention is comparatively less. These observations may be explained by (a) reported high prevalence rates of psychological distress in humanitarian settings [4, 5, 40]; (b) regression to mean phenomena. Natural remission seems to be more frequent in mild and moderate cases of depression [1, 37, 41]; and (c) exclusion of patients with severe symptoms of CMDs and in need of in-patient care from the present study.

## Limitations

This study demonstrates that the addition of PM + intervention to routine care resulted in better treatment outcomes. However, there are a number of limitations which should be borne in mind when interpreting the study findings. First, we were able to follow only 67% of the sample at 20 weeks after baseline. This high dropout rate is expected in public mental health facilities and was accounted for in sample size calculations. Second, while the literature reports long term gains of combined treatment, we were able to follow-up patients for only 20 weeks. Third, we collected data on the use of psychotropic medicines using the CSRI, which may be subject to recall bias of study participants. Further studies may benefit from including larger sample size, longer term follow-up and objective data on the use of psychotropic medications (such as an audit of hospital medical records). Lastly, the mean number of sessions attended by participants in the intervention arm was 2.86. Although, this is in keeping with compliance rates observed in the psychiatric outpatient department in public mental health facilities in low resource settings, this may raise concerns about the adequacy of the dose of intervention received by the trial participants. A meta-regression analysis conducted by Cuijpers et al. (2013) observed a small correlation between number of therapy sessions and treatment effects and this association was not significant when the analysis adjusted for other characteristics of the studies [42]. This evidence suggests that even few but meaningful clinical interactions can benefit symptom improvement in patients with CMDs.

## Conclusions

Results of the study demonstrate that the addition of PM + intervention to routine care resulted in better treatment outcome for the symptoms of anxiety and depression and improved functioning among outpatient attendees of a specialized mental health care facility in Pakistan. Specialized care facilities in LMICs may consider adding brief, evidence-based psychological treatments for CMDs to their routine care.

## Data Availability

The dataset generated and analyzed during the current study are available from the corresponding author on reasonable request.

## Abbreviations

CBT: Cognitive Behavioral Therapy
CMDs: Common Mental Disorders
CSRI: Client Service Receipt Inventory
GHQ-12: General Health Questionnaire-12
HADS: Hospital Anxiety and Depression Scale
IoP: Institute of Psychiatry (IoP)
ITT: Intent-To-Treat
LMICs: Low and middle-income countries
MSPSS: Multidimensional Scale of Perceived Social Support
PCL-C: PTSD Checklist-Civilian version
PHQ-9: Patient Health Questionnaire
PM +: Problem Management Plus
PSYCHLOPS: Psychological Outcome Profiles instrument
PTSD: Posttraumatic Stress Disorder
RCT: Randomized Controlled Trial
TAU: Treatment-As-Usual
WHO: World Health Organization
WHODAS 2.0: WHO Disability Assessment Schedule 2.0
